# P-1008. Polymerase Chain Reaction Cycle Threshold as a Predictor for Cell Culture Cytotoxicity Assay Results in Hospitalized Adults With PCR-Positive Clostridioides Difficile Infection

**DOI:** 10.1093/ofid/ofaf695.1205

**Published:** 2026-01-11

**Authors:** Danh Phan, David T Adams, Satwinder S Kaur

**Affiliations:** Texas Health Resources, Fort Worth, TX; Texas Health Harris Methodist Hospital Fort Worth, Fort Worth, TX; Texas Health Harris Methodist Hospital Fort Worth, Fort Worth, TX

## Abstract

**Background:**

Diagnosing *Clostridioides difficile* infection remains challenging due to high sensitivity but limited specificity of polymerase chain reaction (PCR) assay, which often detect colonization rather than true infection. Alternative testing with improved specificity like the cell culture cytotoxicity assay (CCCA), the gold standard for toxin confirmation, remains time intensive. Emerging evidence suggests PCR cycle threshold (C_T_) values may predict toxin presence. This study explores the relationship between C_T_ values and CCCA results to improve diagnostic accuracy and guide treatment decisions.

**Methods:**

This retrospective cohort study analyzed adults with both *C. difficile* PCR and CCCA test results available at multiple Texas Health Resources hospitals between April 2023 and December 2024. Patients receiving *C. difficile* treatment prior to specimen collection or with incomplete data were excluded. Demographic, laboratory, and clinical data were collected from electronic health records. PCR C_T_ values and CCCA results were assessed using ROC curve analysis to determine diagnostic performance. Sensitivity, specificity, positive predictive value (PPV), and negative predictive value (NPV) were analyzed at different C_T_ thresholds.

**Results:**

A total of 456 samples from 417 unique patients were included; the median age was 68 years, and 33.5% were male. Most patients were classified as community-acquired (82.5%). The median PCR C_T_ value was 27.8. ROC curve analysis yielded an AUC of 0.288, indicating poor predictive performance. As C_T_ values increased, sensitivity and NPV increased, while specificity and PPV decreased. No definitive C_T_ cutoff was identified. Overall, 55.7% of PCR-positive specimens were confirmed positive by CCCA.

**Conclusion:**

Our preliminary findings suggest a potential relationship between lower C_T_ values and CCCA positivity, but no definitive C_T_ cutoff was identified. The trends observed in NPV and PPV of C_T_ values may reflect differences in bacterial burden, but interpretation is currently limited by small sample size. While C_T_ values may provide contextual value in diagnostic interpretation, they remain investigational and should not guide clinical decisions alone.

**Disclosures:**

All Authors: No reported disclosures

